# Bioclimatic variables dataset for baseline and future climate scenarios for climate change studies in Hawai'i

**DOI:** 10.1016/j.dib.2022.108572

**Published:** 2022-09-02

**Authors:** Lucas Berio Fortini, Lauren R. Kaiser, Lulin Xue, Yaping Wang

**Affiliations:** a U.S. Geological Survey, Pacific Island Ecosystems Research Center, Honolulu, HI, USA; b Hawai'i Cooperative Studies Unit, Honolulu, HI, USA; c National Center for Atmospheric Research, Boulder, CO, USA

**Keywords:** Bioclimatic Variables, Regional Projections, GCMs, Climate Shifts, Future Scenarios

## Abstract

Gridded bioclimatic variables representing yearly, seasonal, and monthly means and extremes in temperature and precipitation have been widely used for ecological modeling purposes and in broader climate change impact and biogeographical studies. As a result of their utility, numerous sets of bioclimatic variables have been developed on a global scale (e.g., WorldClim) but rarely represent the finer regional scale pattern of climate in Hawai'i. Recognizing the value of having such regionally downscaled products, we integrated more detailed projections from recent climate models developed for Hawai'i with current climatological datasets to generate updated regionally defined bioclimatic variables. We derived updated bioclimatic variables from new projections of baseline and future monthly minimum, mean, and maximum temperature (T_min_, T_mean_, T_max_) and mean precipitation (P_mean_) data at 250 m resolution. We used the most up-to-date dynamically downscaled projections based on the Weather Research and Forecasting (WRF) model from the International Pacific Research Center (IPRC) and the National Center for Atmospheric Research (NCAR). We summarized the monthly data from these two climate projections into a suite of 19 standard bioclimatic variables that provide detailed information about annual and seasonal mean climatic conditions for the Hawaiian Islands. These bioclimatic variables are available for three climate scenarios: baseline climate (1990-2009) and future climate (2080-2099) under representative concentration pathway (RCP) 4.5 (IPRC projections only) and RCP 8.5 (both IPRC and NCAR projections) climate scenarios. The resulting dataset provides a more robust set of climate products that can be used for modeling purposes, impact studies, and management planning.

**Specifications Table**

**Table:** 

Subject	Ecological Modelling
Specific subject area	Bioclimatic variables for local climate change impact studies in the Hawaiian Islands
Type of data	GeoTIFF spatial datasets
How the data were acquired	**Observation-based baseline data-** We used 250 m resolution observation-based monthly P_mean_ from the Rainfall Atlas of Hawai'i [1] and monthly T_min_, T_mean_, T_max_ from the Climate of Hawai'i [2] datasets as our closest estimate of baseline temperature and precipitation patterns across the archipelago. **Monthly grids from IPRC HRCM Regional Projections-** The IPRC Hawai'i Regional Climate Model (HRCM) is a WRF dynamic downscaling model configured for the Hawaiian Islands [3], [4], [5]. We used the most recently updated HRCM products for baseline and future projections. The HRCM baseline period is 1990-2009, with projections available at 1-km resolution for all the major Hawaiian Islands. Future projections are available for end-of-century (2080-2099) conditions under RCP 4.5 and 8.5. Temperature and precipitation variables were downloaded from the U.S. Geological Survey Center for Integrated Data Analytics catalog (USGS CIDA https://cida.usgs.gov/thredds/catalog.html) online. **Monthly grids from NCAR WRF Regional Projections-** Recently developed fine-scale WRF regional climate simulations by NCAR provide 10-year baseline (2002-2012) and future scenarios (2090-2100, RCP 8.5 only) for Hawai'i [6]. Monthly gridded baseline and future estimates of P_mean_, and T_min_, T_mean_, and T_max_ variables were obtained from NCAR.
Data format	Analyzed
Description of data collection	**Aligning baseline periods between baseline and DD model projections-** The differences in baseline periods between the observational data and the dynamical downscaling (DD) baseline projections make their integration impossible without standardization. For each DD baseline projection, we used different overlapping periods from the observational data per variable to standardize the baseline period. We then adjusted the HRCM and NCAR DD baseline projections so that their baseline periods aligned with available observational data. **Future Climate Projections-** We relied on the standard delta method for bias correction [7] to reduce the effect of baseline deviations in the spatial pattern of temperature and precipitation from HRCM projections. We calculated the percent change in precipitation and the absolute change of temperature (in degrees). We then applied these calculated changes to the aligned observational data across all months. These bias corrected calculations were done for the future HRCM (2080-2099) and NCAR (2090-2100) projections for all variables (P_mean_, T_min_, T_mean_, T_max_).
Data source location	Main Hawaiian Islands, state of Hawai'i, USA.
Data accessibility	The datasets generated during and/or analysed during the current study are available in the USGS repository, https://doi.org/10.5066/P9MF7SG. Repository name: USGS ScienceBase Catalog Data identification number: Direct URL to data: https://doi.org/10.5066/P9MF7SG

## Value of the Data

• To account for the inherent uncertainty of future climate shifts, multiple climate projections are needed for reliable climate change impact studies. In Hawai'i, obtaining such reliable climate data products has been a challenge due to the wide range of climate gradients, complex topography, and the necessary fine spatial scale required to reliably represent the island archipelago.
• In recognizing the utility and value of having regionally downscaled products, we integrated detailed projections from recent climate models developed for Hawai'i with current climatological datasets to generate regionally defined bioclimatic variables available at 250 m resolution for baseline climate (1990-2009) and future climate (2080-2099) under RCP 4.5 and RCP 8.5 climate scenarios.
• The provided bioclimatic variables describe temperature and rainfall variability, as well as potential changing interactions between the two. Using the multiple future scenarios, we can estimate the changes of the individual bioclimatic variables when compared to the baseline scenario to determine the direction and amount of change.
• Because annual rainfall in most areas in Hawai'i is characterized by two 6-month seasons, we also provide mean seasonal variables for all scenarios based on the dry (May-October) and wet (November-April) seasonality of Hawaiian climate.
• Differences in future projections in this dataset partially illustrate the variability of possible scenarios that could be realized in the future. These new climatic datasets can be used, along with other available climate projections, to better represent the future uncertainty in climate-related studies in Hawai'i.
• These bioclimatic variables can be key when explaining the current distribution and predicting future variation in species richness under a changing climate [8], [9], [10], [11], [12] and are also relevant to a wider range of studies as they can be used to better understand trends in human health, agriculture, and more [13], [14], [15], [16].

## 1 Data Description

General circulation models (GCMs) offer a sophisticated representation of the general climate system and inform future projections at the global scale. However, GCMs are typically at such a coarse resolution that the models do not reproduce the fine-scale spatial patterns of climate in Hawai'i [17]. This leaves land and natural resource managers with limited resources to inform adaptive management processes and future conservation plans. As a result, GCM projections have been downscaled to better represent climate at more refined spatial scales relevant to management and decision making [18].

Globally, bioclimatic variables are widely used in species distribution modeling and in broader climate change impact studies and biogeographical studies. A literature search for ‘bioclimatic variables’ & ‘distribution models’ alone yields > 3,500 publications. These are datasets so commonly used in climate impact studies that now multiple research groups provide similar global bioclimatic variable datasets such as WorldClim 2, CHELSA, MERRAclim, ecoClimate and others [19], [20], [21], [22]. However, these global datasets poorly represent the Hawaiian regional climate and fine scale patterns such as orographically determined rainfall and the tradewind inversion [23]. Recognizing the value of regionally downscaled climate projections in replicating such regional climatic patterns, past efforts generated a regionally derived bioclimatic dataset for Hawai'i in 2015 [24], with the resulting variables being widely used in Hawai'i for climate impact studies and other biogeographical studies [25], [26], [27]. As useful as those regional bioclimatic datasets have been, their future projections are based on a single outdated SRES emission scenario (A1B) and on older CMIP3 global circulation models.

Bioclimatic variables are biologically meaningful indicators that describe how climate affects ecosystems and services. They are derived from monthly temperature and rainfall values that then represent annual and seasonal climatic trends. Recognizing the value of regionally downscaled climate projections in replicating regional climatic patterns found in Hawai'i, we integrated updated fine scale IPRC HRCM [5] and new NCAR [6] projections with observation-based precipitation and temperature datasets to project updated regionally defined bioclimatic variables for Hawai'i. We generated a revised set of bioclimatic variables at 250 m resolution for a baseline climate and future climate scenarios under RCP 4.5 and RCP 8.5 scenarios.

This bioclimatic data series provides continuous rasters for 19 predictor variables (Table 1, https://doi.org/10.5066/P9MF7SG) that highlight climatic conditions for the State of Hawai'i under both baseline and end-of-century (RCP 4.5 and RCP 8.5) scenarios. These bioclimatic variables provide detailed information about annual conditions (annual mean temperature, annual precipitation, annual range in temperature and precipitation), as well as seasonal mean climate conditions (temperature of the coldest and warmest months, precipitation of the wettest and driest quarters). Each of these bioclimatic variables are available for one baseline scenario and three projected future scenarios. The baseline scenario provides an estimate of current (1983-2012) climatic conditions for each bioclimatic indicator. Future IPRC (2080-2099) and NCAR (2090-2100) projections are available for one RCP 4.5 scenario (IPRC projection) and two RCP 8.5 scenarios (IPRC and NCAR projections). From these multiple future scenarios, we can estimate the changes of the individual bioclimatic variables compared to the baseline scenario to determine the direction and amount of change. Having these multiple projections offers more variability in the potential climatic changes that may be realized in the future across all the Hawaiian Islands.

**Table 1:** List of Bioclimatic Variables.

Bioclimatic variable	Description
1	Annual mean temperature
2	Mean diurnal range (Mean of monthly max temperature - min temperature)
3	Isothermality (Mean diurnal range/ temperature annual range)
4	Temperature seasonality (Standard deviation of monthly mean temperature)
5	Max temperature of warmest month
6	Min temperature of coldest month
7	Temperature annual range (Max temperature of warmest month - min temperature of coldest month)
8	Mean temperature of wettest quarter
9	Mean temperature of driest quarter
10	Mean temperature of warmest quarter
11	Mean temperature of coldest quarter
12	Annual precipitation
13	Precipitation of wettest month
14	Precipitation of driest month
15	Precipitation seasonality (Coefficient of variation for monthly precipitation)
16	Precipitation of wettest quarter
17	Precipitation of driest quarter
18	Precipitation of warmest quarter
19	Precipitation of coldest quarter

**Fig. 1:**
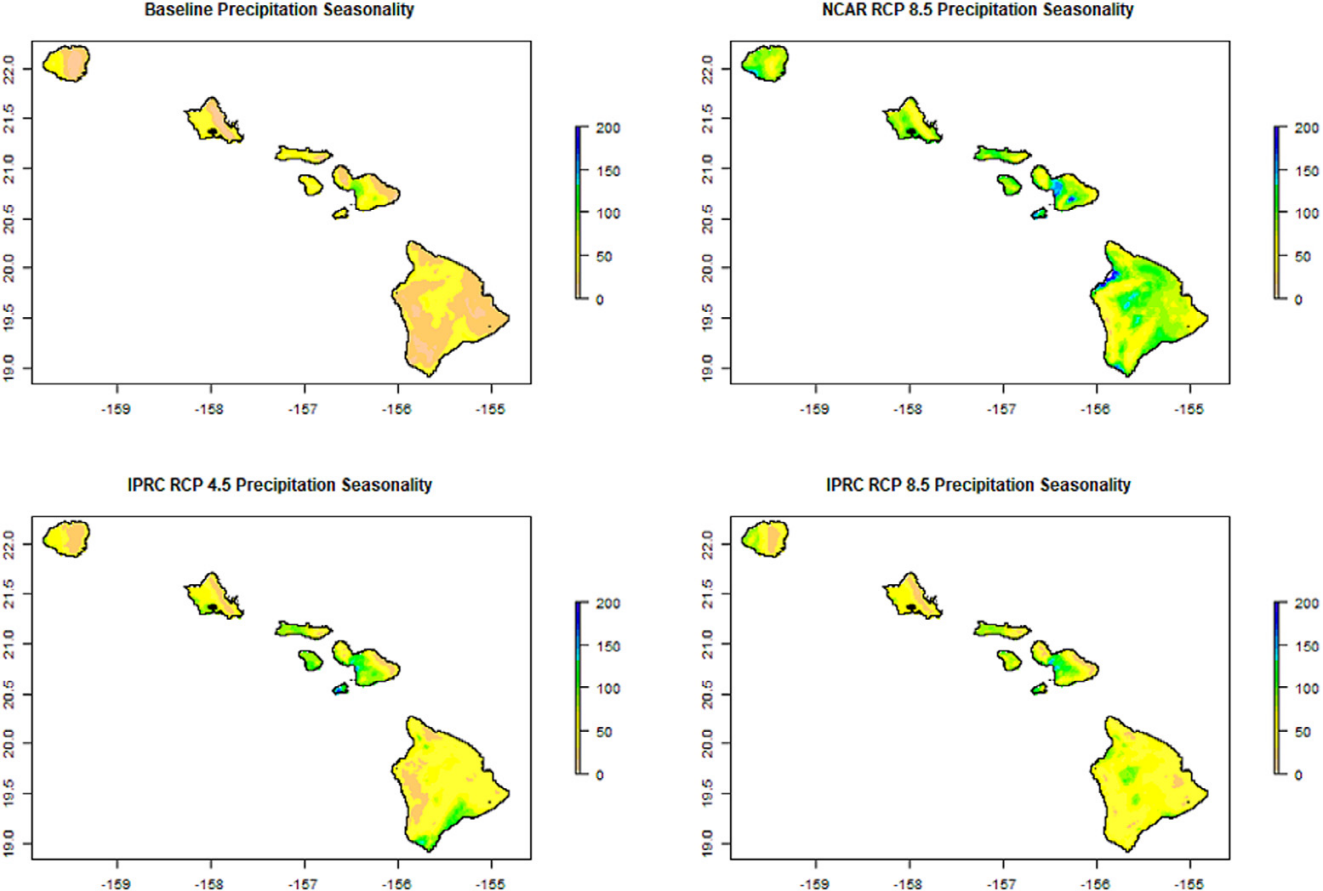
Example Bioclimatic Variable. The a) baseline and the projections by b) NCAR (RCP 8.5) and c-d) IPRC (RCP 4.5 and RCP 8.5, respectively) for Precipitation Seasonality (as %) (BIO 15).

**Fig. 2:**
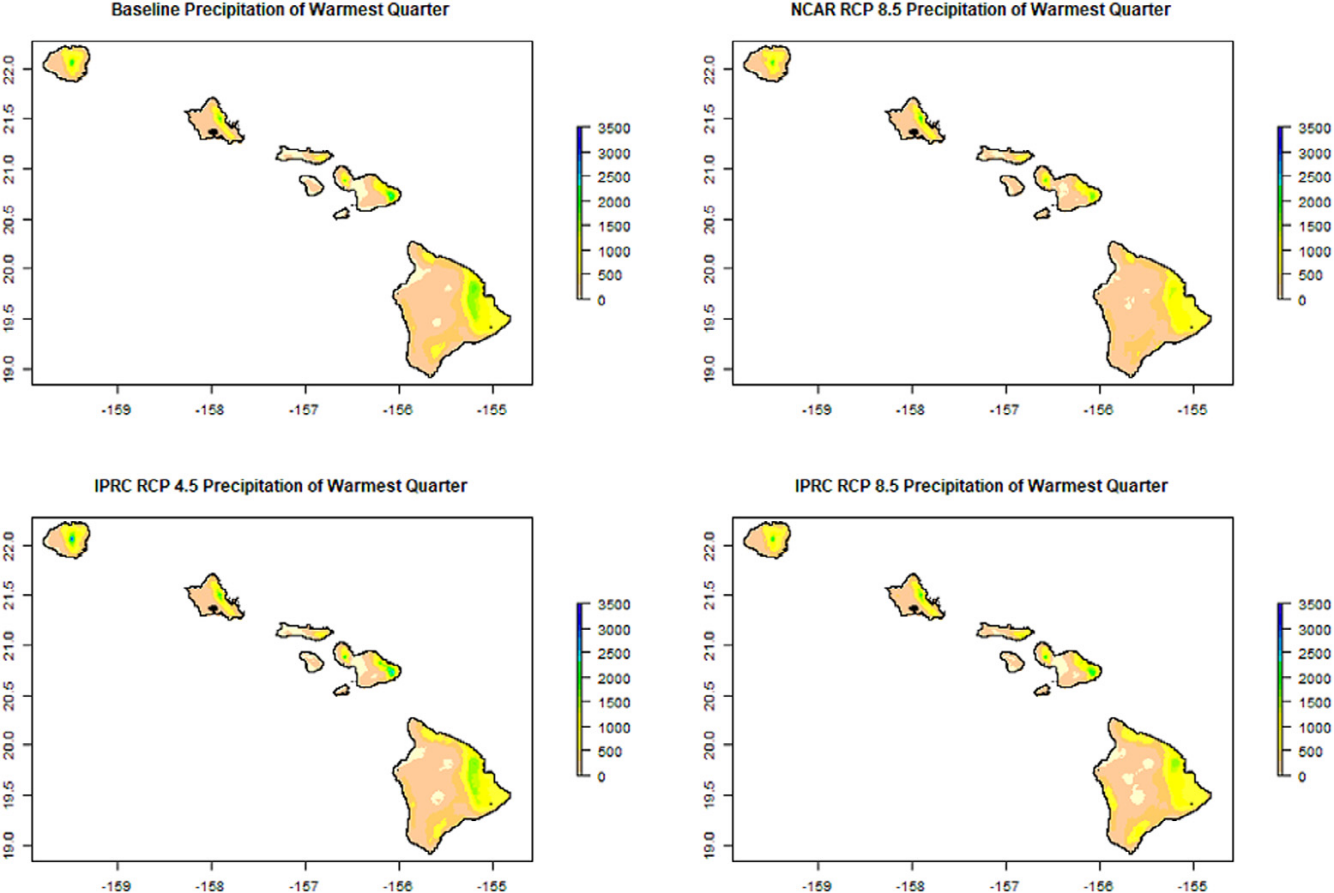
Example Bioclimatic Variable. The a) baseline and the projections by b) NCAR (RCP 8.5) and c-d) IPRC (RCP 4.5 and RCP 8.5, respectively) for Precipitation of the Warmest Quarter in mm (BIO 18).

These bioclimatic variables describe changes in temperature and rainfall variability, as well as potential changing interactions between the two. For instance, Precipitation Seasonality (BIO 15) shows the projected shifts in rainfall variability (Fig. 1). Other bioclimatic indices describe interactions between rainfall and temperature, such as Precipitation of the Warmest Quarter (Fig. 2). This index estimates precipitation that falls during the warmest three months of a year, which can be useful to characterize critical drought stress periods and seasonal distributions of species.

**Fig. 3:**
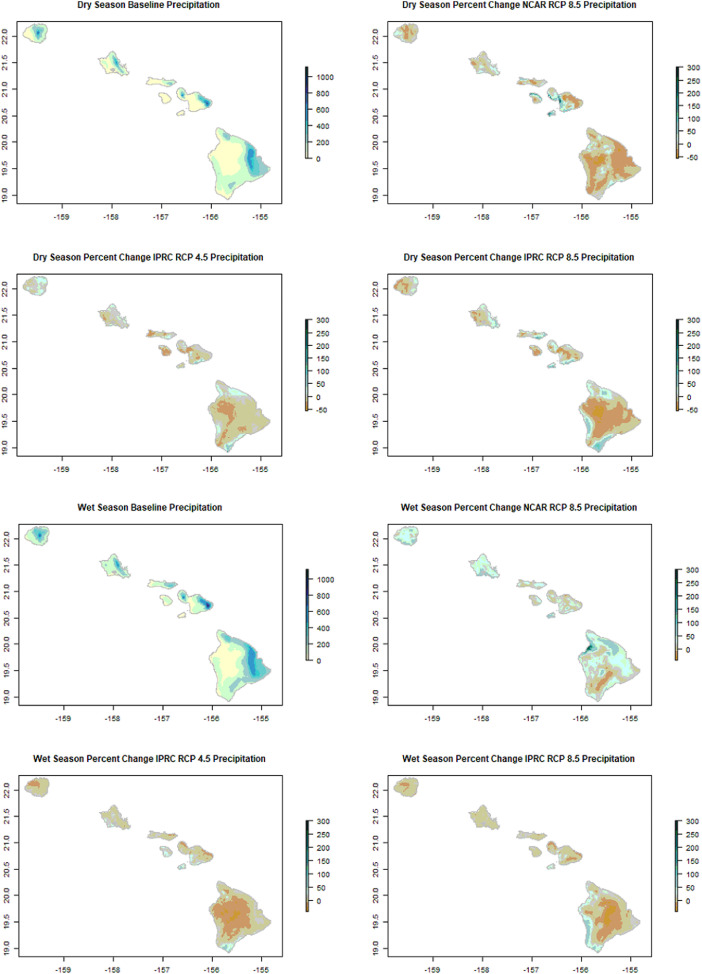
Percent Change in Dry and Wet Season Precipitation. The a,e) baseline mean precipitation in mm and percent (%) change for b,f) NCAR RCP 8.5, c,g) IPRC RCP 4.5, and d,h) IPRC RCP 8.5 projections for dry and wet seasons, respectively.

Aside from the typical bioclimatic variables, we also calculated mean seasonal variables for all scenarios based on the dry (May-October) and wet (November-April) seasonality of Hawaiian climate (Fig. 3). Because annual rainfall in most areas in Hawai'i is characterized by these two 6-month seasons, potential shifts of these seasonal phases are important to consider.

## 2 Experimental Design, Materials and Methods

Gridded monthly mean precipitation (P_mean_) and monthly min (T_min_), mean (T_mean_), max (T_max_) temperature data are required for calculation of standard bioclimatic indicators. We used observation-based data for the baseline bioclimatic variables and two dynamical downscaling (DD) projections for two future scenarios. We describe how these datasets for current baseline and projected future climate were processed and calculated below.

**Observation-based baseline data-** We used 250 m resolution observation-based monthly P_mean_ from the Rainfall Atlas of Hawai'i [1] and monthly T_min_, T_mean_, T_max_ from the Climate of Hawai'i [2] datasets as our closest estimate of baseline temperature and precipitation patterns across the archipelago. These two datasets have differing historical periods, with the observation-based mean annual precipitation data representing a historical period from 1978–2007 and annual temperature data representing a historical period from 1957-1980. These precipitation and temperature datasets are considered the best available representation of the current baseline climate across the islands and thus are widely used in Hawai'i.

**Regional downscaling projections-** Currently in Hawai'i, there are few efforts to regionally downscale global models. Elison Timm et al. [28,29] generated statistical downscaling (SD) climate projections of precipitation and temperature for Hawai'i by developing a statistical relationship between regional-scale spatial patterns of atmospheric circulation, moisture transport, and stability and point-scale observations. Efforts from the International Pacific Research Center (IPRC) [3,4] and the National Center for Atmospheric Research (NCAR) [6] have both used DD approaches to generate a higher resolution regional climate model that use pseudo global warming (PGW) [30,31] to determine regional model parameters, such as lateral and boundary conditions. Both DD products are derived using the Weather Research and Forecasting (WRF) model for historical and future scenarios [32]. The IPRC configured a nested version of the WRF model with both high resolution and improved physics for the Hawaiian region, known as the Hawai'i Regional Climate Model (HRCM). Updates and improvements to the configured HRCM include additional details like the specification of surface properties such as albedo, land cover type, and soil type for the Hawaiian Islands. These updated HRCM projections from the IPRC are available for regional climate projections (RCP) 4.5 and 8.5 emission scenarios [33]. The new NCAR projections, based on two 10-year periods, implement change to historical (2002-2012) conditions based on climate change signals from GCM averages under future (2090-2100) RCP 8.5 emissions. These simulations from NCAR have been validated and have well documented results that ensure the reliability and integrity of the data [6].

**Processing of monthly grids from IPRC HRCM Regional Projections-** The HRCM is a WRF dynamic downscaling model configured for the Hawaiian Islands [3], [4], [5]. In general, the dynamical downscaling approach of the HRCM realistically simulates the magnitude and geographical distribution of mean precipitation in Hawai'i and thus is commonly used for local climate impact studies [34], [35], [36]. We used the most recently updated HRCM products for baseline and future projections. The HRCM baseline period is 1990-2009 and the updated projections are available at 1-km resolution for all the major Hawaiian Islands. Future projections are available for end-of-century (2080-2099) conditions under RCP 4.5 and 8.5.

Temperature and precipitation variables were downloaded from the U.S. Geological Survey Center for Integrated Data Analytics catalog (USGS CIDA https://cida.usgs.gov/thredds/catalog.html) online. Hourly gridded data was collected for baseline (present) and future (RCP 4.5 and 8.5) scenarios. Temperature values were aggregated by monthly minimums and maximums (T_min_ and T_max_). For HRCM projections, mean temperature (T_mean_) values were then calculated from the average of the minimum and maximum values. We used the 95th percentile of values to account for outliers and avoid unrealistic T_min_ and T_max_ values. Mean precipitation (P_mean_) values were derived from two rainfall variables. The hourly count of rainfall tipping buckets was multiplied by 100 and then added to the total accumulation precipitation at the grid scale and again aggregated to a monthly temporal scale.

**Processing of monthly grids from NCAR WRF Regional Projections-** A new set of fine-scale climate models for Hawai'i recently developed and released by NCAR uses two 10-year WRF regional climate simulations for baseline and future scenarios [6]. The baseline simulation is based on the ERA-Interim global reanalysis data and observed sea surface temperature from October 2002 to September 2012. This 10-year historical period was selected to represent the hydrologic seasonality of Hawaii and the availability of ultra-high resolution climate data used in this model setup. The future projection uses the PGW method to implement change based on GCM averages from 2090 to 2100. This dataset has a major advantage of providing validated hourly rainfall values for the entire state. Baseline and future (RCP 8.5 only) projections for P_mean_, and T_min_, T_mean_, and T_max_ variables were provided by NCAR [6]. These data were provided at a monthly gridded scale for the main Hawaiian Islands.

**Aligning baseline periods between baseline and DD model projections-** The differences in baseline periods between the observational data and the DD baseline projections make their integration impossible without standardization. Hence, we adjusted the HRCM and NCAR DD baseline projections so that their baseline periods aligned with the commonly used observational data.

To align the DD precipitation baseline projections, we used monthly gridded precipitation datasets available from 1920-2012 [37]. From these monthly datasets, we created monthly precipitation grids matching the differing baseline periods for the Rainfall Atlas observational dataset (1978-2007), the HRCM monthly precipitation dataset (1990-2009), and the NCAR projections (2002-2012). We then calculated the percent precipitation difference between the original observational baseline period (1978-2007) and the two DD baseline periods (1990-2009 and 2002-2012):

$$HRCM\;P_{1978-2007}=HRCM\;P_{1990-2009}\times \left(1+\;\frac{Monthly\;Obs\;P_{1978-2007}-Monthly\;Obs\;P_{1990-2009}}{Monthly\;Obs\;P_{1990-2009}}\right)$$

To standardize the DD precipitation baseline projections, we used absolute difference instead of percent difference. However, because we did not have equivalent monthly temperature grids, we used statewide yearly temperature records [38,39] to calculate the absolute temperature deviation between the original observational period (1957-2010) and the HRCM and NCAR baseline periods (1990-2009 and 2002-2012, respectively). Ultimately, we applied this absolute change as a correction for the observational temperature dataset used in the analysis:

$$NCAR\;T_{1957-2010}=NCAR\;T_{1957-1980}+\left(Yearly\;Obs\;T_{1957-2010}-Yearly\;Obs\;T_{1957-2010}\right)$$

**Future Climate Projections-** We relied on the standard delta method for bias correction [7] to reduce the effect of baseline deviations in the spatial pattern of temperature and precipitation from HRCM projections. We calculated the percent change in precipitation and the absolute change of temperature (in degrees). We then applied these calculated changes to the aligned observational data across all months. These bias corrected calculations were completed for the future HRCM (2080-2099) and NCAR (2090-2100) projections for all variables (P_mean_, T_min_, T_mean_, T_max_):

$$Bias\;corrected\;HRCM\;P_{2080-2099}=Obs\;P_{1978-2007}\times \left(1+\;\frac{HRCM\;P_{2080-2099}-HRCM\;P_{1978-2007}}{HRCM\;P_{1978-2007}}\right)$$

$$Bias\;corrected\;HRCM\;T_{2080-2099}=Obs\;T_{1957-2010}+\left(HRCM\;T_{2080-2099}-HRCM\;T_{1957-2010}\right)$$

**Bioclimatic and seasonal variable calculations-** Once precipitation and temperature rasters were calculated for each of the baseline and future scenarios considered, we calculated the 19 bioclimatic variables using the methods available in the ‘dismo’ R package [40] also used to calculate commonly used WorldClim bioclimatic variables [19]. Table 1 describes each variable and calculation. These methods are based on a dynamic temporal definition of bioclimatic variables where, for instance, BIO14 (the precipitation of driest month) may refer to a different month for baseline conditions as compared to a future projected scenario if rainfall seasonality is projected to change). Past research shows no clear advantage/disadvantage of using dynamic versus static reference months and quarters for bioclimatic variable calculations [41]. We also calculated mean seasonal T_min_, T_max_, T_mean_ and P_mean_ variables for all scenarios considered (RCP 4.5 and RCP 8.5) based on fixed dry (May-October) and wet (November-April) Hawaiian seasons.

## Ethics Statements

The present work did not involve the use of human subjects, animal experiments, or data collected from social media platforms.

## CRediT Author Statement

**Lucas B**erio **Fortini:** Conceptualization, Methodology, Investigation; **Lauren R. Kaiser:** Visualization, Writing – draft preparation; **Lulin Xue:** Data curation, Writing – review & editing; **Yaping Wang:** Data curation, Writing – review & editing.

## Declaration of Competing Interest

The authors declare that they have no known competing financial interests or personal relationships that could have appeared to influence the work reported in this paper.

## Data Availability

Hawaiian Islands bioclimatic variables for baseline and future climate scenarios (Original data) (sciencebase).
